# A Phloem-Expressed *PECTATE LYASE-LIKE* Gene Promotes Cambium and Xylem Development

**DOI:** 10.3389/fpls.2022.888201

**Published:** 2022-04-26

**Authors:** Max Bush, Vishmita Sethi, Robert Sablowski

**Affiliations:** Cell and Developmental Biology Department, John Innes Centre, Norwich Research Park, Norwich, United Kingdom

**Keywords:** Arabidopsis, shoot development, pectate lyase, phloem, cambium, xylem

## Abstract

The plant vasculature plays essential roles in the transport of water and nutrients and is composed of xylem and phloem, both of which originate from undifferentiated cells found in the cambium. Development of the different vascular tissues is coordinated by hormonal and peptide signals and culminates in extensive cell wall modifications. Pectins are key cell wall components that are modified during cell growth and differentiation, and pectin fragments function as signals in defence and cell wall integrity pathways, although their role as developmental signals remains tentative. Here, we show that the pectin lyase-like gene *PLL12* is required for growth of the vascular bundles in the Arabidopsis inflorescence stem. Although PLL12 was expressed primarily in the phloem, it also affected cambium and xylem growth. Surprisingly, PLL12 overexpression induced ectopic cambium and xylem differentiation in the inflorescence apex and inhibited development of the leaf vasculature. Our results raise the possibility that a cell wall-derived signal produced by *PLL12* in the phloem regulates cambium and xylem development.

## Introduction

The vasculature of land plants is critical for growth, as it distributes water and nutrients throughout the plant and provides mechanical support (Ruonala et al., 2017). Each vascular bundle contains three types of tissues: xylem, including water-transporting xylem elements, the mechanically reinforced xylem fibres and parenchyma; phloem, containing the sugar-transporting sieve elements, the companion cells that regulate movement of molecules in and out of the phloem, phloem fibres and parenchyma; and cambium, a layer of undifferentiated cells that produces new xylem cells toward the plant’s main axis and new phloem cells away from it (Nieminen et al., 2015).

During the early stages of vascular development, the position of xylem, phloem and the procambium (precursor of the cambium) is defined by intercellular signals mediated by the auxin and cytokinin hormones, in combination with mobile transcription factors and microRNAs (Carlsbecker et al., 2010; Miyashima et al., 2019). The subsequent production of new vascular cells by the cambium is regulated by the peptide signals CLAVATA3/EMBRYO SURROUNDING REGION (CLE) CLE41/CLE44, which activate the Phloem Intercalated With Xylem (PXY) receptor to regulate the balance between the production of new phloem and xylem cells (Ito et al., 2006; Fisher and Turner, 2007; Hirakawa et al., 2008). The ensuing differentiation of these tissues is guided by cell type-specific transcription factors, such as *VASCULAR-RELATED NAC DOMAIN 6* (*VND6*) and *VND7*, which specify xylem cell types (Kubo et al., 2005), or Altered Phloem Development (APL) (Bonke et al., 2003) and *NAC DOMAIN-CONTAINING PROTEIN 45* (*NAC45*) (Furuta et al., 2014), which control phloem differentiation.

Among the processes regulated during vascular differentiation, modifications of the cell wall feature prominently, for example in the deposition of lignified hoops that mechanically reinforce xylem elements (Mitsuda et al., 2007; Sugiyama et al., 2017), or in the development of perforated plates that connect adjacent sieve elements (Kalmbach and Helariutta, 2019). Pectic polysaccharides, of which the most abundant form is homogalacturonan, are important components of the plant cell wall that are modified during cell growth and differentiation. Pectins are secreted in a methyl-esterified form and modified by pectin methylesterase (PME) to control the degree of methylesterification. Pectins with low levels of methylesterification bind Ca^2+^ within the cell wall to form a cross-linked gel that surrounds cellulose and is believed to decrease cell wall extensibility (Wolf et al., 2012; Yang et al., 2018). Developmentally controlled pectin methylesterification modulates not only wall extensibility, but also access to further cell wall-modifying enzymes, such as those involved in the deposition of lignin during xylem differentiation (Wolf et al., 2012).

One group of cell wall modifiers that target demethylesterified pectin are the PECTATE LYASE-LIKE (PLL) proteins, which are found throughout the plant kingdom and are encoded by large gene families (Uluisik and Seymour, 2020). PLLs have an N-terminal signal peptide that targets them for secretion into the cell wall, where they cut homogalacturonan at β(1–4) linkages to generate oligogalacturonides (OGs) of different sizes (Sénéchal et al., 2014; Yang et al., 2018). Pectin cleavage occurs during cell expansion or remodelling, and in the middle lamella during cell separation (Yang et al., 2018). Accordingly, PLLs have been implicated in cell expansion processes, such as elongation of cotton fibres (Sun et al., 2020), in secondary wall formation in the xylem, and in cell separation during fruit ripening (Uluisik and Seymour, 2020). Additionally, the OGs produced during pectin cleavage have a signalling role, studied mostly in relation to pathogen attack and as part of the cell wall integrity pathway (Brutus et al., 2010; Ferrari et al., 2013). Externally applied OGs also have developmental consequences, for example by inhibiting the effect of auxin on pea stem elongation (Branca et al., 1988), although a role for OGs as endogenous developmental signals still remains to be established (Ferrari et al., 2013).

Here, we characterise the function of *PLL12* in Arabidopsis vascular development. We selected this gene based on ChIP-seq data for the BEL1-like homeodomain transcription factor REPLUMLESS (RPL) (also known as PENNYWISE, PNY and BELLRINGER, BLR) (Byrne et al., 2003; Roeder et al., 2003; Smith and Hake, 2003), which is required for correct vascular patterning (Smith and Hake, 2003; Etchells et al., 2012) and directly interacts with multiple genes that regulate vascular development, such as *PXY*, *CLE41* and *NAC45* (Bencivenga et al., 2016). Furthermore, RPL has been implicated in the expression of cell wall enzymes involved in vascular differentiation (Peaucelle et al., 2011; Etchells et al., 2012). We reasoned that less well-characterised RPL target genes, such as *PLL12*, might reveal novel aspects of vascular development. Our functional analysis showed that *PLL12* is expressed in the phloem but has unexpected cell non-autonomous effects on cambium and xylem development.

## Materials and Methods

### Plant Material

Plants were grown on JIC *Arabidopsis* Soil Mix (Levington F2 compost plus Intercept and 4 mm grit at a 6:1 ratio) at 16°C under continuous light (100 mE) or in a Sanyo cabinet at 18°C under 16 h light/8 h dark cycles (100 mE). *Arabidopsis thaliana* Columbia (Col) and Landsberg-*erecta* (L-*er*) were used as wild-types; *pll12* (AT5g04310; SAIL 1149_C06) was obtained from The Nottingham Arabidopsis Stock Centre and genotyped using primers 1–3 Table 1. For the construction of RPS5A:LhGR:op*PLL12*, the *PLL12* CDS was amplified from Col cDNA (primers 12–13 Table 1), cloned into pGEMT-EASY, sequenced, moved into pOWL49 by *Sal*I/*Kpn*I restriction cloning and transformed into *RPS5A:LhGR* in L-*er* background. Transformants were selected on gentamycin/kanamycin Murashige and Skoog medium (M&S) with agar, transferred to soil and treated at appropriate developmental stages with either 10 μM dexamethasone (from a 10 mM stock in ethanol) or the equivalent volume of ethanol (mock-treatment) diluted in aqueous 0.015% Silwet L-77 (49). Some seedlings were germinated and grown on M&S agar and then transferred to M&S agar supplemented with either 10 μM dexamethasone or ethanol (mock).

**TABLE 1:** Oligonucleotide sequences.

Number	Name	Sequence
**Genotyping**		
1	PLL12 F	TCATTTTCATGCTTTATCTTGTG
2	PLL12 R	CCTGACTATTAATTGTAGGGCTA
3	SAIL LB1 TDNA	GCCTTTTCAGAAATGGATAAATAGCCTTG CTTCC
**RT-PCR**		
4	PLL12 RTPCR F1	ATGGTGGCTCATGAGAGGAGGATCC
5	PLL12 RTPCR R1	CTATGATCTCGTATGGTGTGGAATATA
6	PLL12 RTPCR R2	CTTTGAACATTAAGAACAACATGTTC
7	PLL12 RTPCR R3	TCGTCGTGACCTAGGAGCATAACC
**qPCR**		
8	PLL12 qPCR F	TCCCAATGCCAAAGAGGTAACG
9	PLL12 qPCR R	TCCAGTTCCATCCCGACCAATG
10	TUBULIN4 qPCR F	CTGTTTCCGTACCCTCAAGC
11	TUBULIN4 qPCR R	AGGGAAACGAAGACAGCAAG
**pOp:PLL12**		
12	PLL12 CDS F	CTGTCGACATGGTGGCTCATGAGAGGAGG
13	PLL12 CDS R	CTGGTACCCTATGATCTCGTATGGTGTGG
***pPLL12:PLL12***		
14	pPLL12 fragment 1 F	GTGGTCTCA*GGAG*ACCTGGTCTGGTTTC ATCAGC
15	pPLL12 fragment 1 R	GTGGTCTCA*GAAA*TCGAATCAATGAAACT CTTG
16	pPLL12 fragment 2 F	GTGGTCTCG*TTTC*TTTAGTGAAGATACA TTTG
17	pPLL12 fragment 2 R	GTGGTCTCA*CATT*ATATCTCCTATTATATC TTATG
18	PLL12 gene fragment 1 F	GTGGTCTCT*AATG*GTGGCTCATGAGA GGAGG
19	PLL12 gene fragment 1 R	GTGGTCTCT*GATG*AGAGAAATAGT TGTTG
20	PLL12 gene fragment 2 F	GTGGTCTCG*CATC*ACGACGAGGT TATGC
21	PLL12 gene fragment 2 R	GTGGTCTCG*GCAT*TGACCGTGAGC TGGTC
22	PLL12 gene fragment 3 F	GTGGTCTCA*ATGC*CGGCGTTTTCGG CGATC
23	PLL12 gene fragment 3 R	GTGGTCTCA*CTTG*CTAAAGAATGCT AAAC
24	PLL12 gene fragment 4 F	GTGGTCTCG*CAAG*TTGGTATATCTCAA AAAC
25	PLL12 gene fragment 4 R	GTGGTCTCT*AAGC*CTATGATCTCGTATG GTGTGG
26	PLL12 3′utr F	GTGGTCTCA*GCTT*TTTCATTATTGGTTCATA GTTAC
27	PLL12 3′utr R	GTGGTCTCA*AGCG*AAGGGAAATCCTGA ATTGACTTG
***PXY*** ** RNA *in situ* hybridisation**		
28	PXY F *in situ*	AAGGATCCATCAGCAATAACCTCTCAGG TGAAG
29	PXY R *in situ*	AAGTCGACTGCATCCAACGTAATTTGGGA TTTCAC

*Sequences re shown in 5′–3′ orientation. Restriction enzyme sites are underlined, Golden Gate recombination overhangs are shown in italics.*

To complement the *pll12* phenotype, a full length p*PLL12:PLL12* construct was generated as follows. Using Columbia genomic DNA and Phusion polymerase (New England Biolabs), the promoter and 5′ utr (1,884 bp) were amplified as two fragments, the gene (3,997 bp) as four fragments and the 702 bp 3′ utr as a single fragment using primers 14–27 in Table 1. The seven fragments were assembled into plasmid G45 by Golden Gate cloning (Engler et al., 2014). The assembly was verified by sequencing and inserted into plasmid G800 by Gateway cloning. For construction of p*PLL12:PLL*:*GUS*, *PLL12* was amplified from Col-0 genomic DNA and fused in-frame with GUS and cloned into pPZP222 (Hajdukiewicz et al., 1994) using Golden Gate cloning as above, with primers listed in Table 1. The final constructs were transformed into Col-0 by the floral dip method (Clough and Bent, 1998) and transformants were selected on Murashige and Skoog medium supplemented with gentamycin 100 μg/mL.

### Measurements of Stem Growth

*Arabidopsis* Col and *pll12* plants were grown until the first flower reached stage 17 (Smyth et al., 1990), ink marks were placed on the stem at 2 mm intervals and photographed next to a ruler using a Nikon D3100 DSLR camera and 18–55 mm VR lens. Plants were returned to the growth cabinet for 4 d and then re-photographed. Manual land marking of the stem ink marks and calibration points on the ruler was performed using the Point Picker plugin of Fiji (Schindelin et al., 2012). Digitalised coordinates were measured, graphs were plotted, and Mann-Whitney U tests and Student’s t tests were performed using standard functions in matplotlib,^1^ Python 2.7, and Scientific Python.^2^

### Microscopy and Staining

Expression of GUS reporters in stem tissue was performed as described (Sieburth et al., 1998), stained tissue was observed with a Leica S8APO stereozoom or Zeiss Axio Imager Z2. Modified pseudo-Schiff propidium iodide (mPS-PI) staining was performed before imaging samples with a Zeiss LSM780 confocal microscope as described (Serrano-Mislata et al., 2015). Staining with 0.04% Calcofluor was performed on Technovit sections for 5 min at room temperature.

To determine if xylem bundles in *pll12* stems were continuous and intact, 1 cm stem apices were stained with propidium iodide (Banasiak et al., 2019) and examined using a Zeiss Axiozoom V16 microscope.

Tissues for light microscopy were fixed in either 1% glutaraldehyde in 0.05 M cacodylate buffer pH 7.2 or formalin:acetic acid:ethanol (4:5:50), dehydrated in an ethanol series and embedded in Technovit following the manufacturer’s instruction. Sections were cut 5 μm thick with glass knives and a Leica ultramicrotome UC7, stained with toluidine blue and examined with a Zeiss Axio Imager Z2 microscope.

Stems for vibratome sectioning were prepared following the protocol of Wang et al. (2014), sections were cut 25–30 μm thick using a Leica VT1000S vibratome, stained with phloroglucinol-HCl (Mitra and Loqué, 2014) and mounted on glass slides following Speer’s modification (Speer, 1987).

### RNA *in situ* Hybridisation Experiments

Stem apices of *RPS5A:LhGR:opPLL12* plants were treated with or without dexamethasone: all stem apices were fixed and subsequently processed according to Rebocho et al. (2017). Serial sections from control (mock-treated) and experimental (dex-treated) samples were collected onto the same Poly-L-Lysine coated slide. Duplicate slides were sandwiched together between plastic spacers and loaded with either sense or antisense probes. To generate specific digoxigenin-labelled riboprobes probes, 550 bp of PXY coding sequence was cloned using primers 28-29 (Table 1) and BamH1/Sal1 ligated into pBluescript II KS9 (±). Antisense probes were PCR amplified using combinations of M13R primer and the specific gene F primer, whilst sense probes were amplified similarly using M13F and specific gene R primers. Purified antisense and sense probes were obtained by RNA transcription using the T3 and T7 promoters respectively following the protocol of Rebocho et al. (2017).

### Rhodamine B Uptake

Col and *pll12* plants grown to the same developmental stage (3–5 flowers/siliques) were removed from their pots and very gently washed under running tap water to remove soil from the root balls before blotting dry with absorbent paper towels. Roots were then threaded through holes cut into a disc of polystyrene foam and the plants floated in a glass beaker containing 50 ml of 0.25% rhodamine B (w/v) so that the roots were immersed in the solution. Batches of 3–4 Col and *pll12* plants were incubated simultaneously under a 6 h light-8 h dark-6 h light regime at 16°C in a Sanyo cabinet. The following day, excess dye was rinsed off the roots which were then blotted dry and the plants photographed and stem samples collected. All but the smallest flower buds were removed before the apical 1 cm and basal 1 cm of stem above the rosette were isolated, frozen in liquid nitrogen and stored at −70°C. Individual stem samples were ground up in 400 μl protein extraction buffer (100 mM HEPES pH 7.5, 5% v/v glycerol, 50 mM KCl, 5 mM EDTA, 0.1% v/v Triton X-100, 1 mM DTT and one Complete EDTA-free protease inhibitor cocktail tablet/50 ml buffer) and rotated at 4°C for 1 h to extract soluble proteins and the rhodamine B dye. After a 10 min centrifugation at 12,000 g to pellet insoluble material, 100 μl aliquots of the extracts were collected into wells of a 96-well microtitre plate and assayed spectrophotometrically at A_550_nm; dye concentrations were calculated against a rhodamine B standard curve. Soluble proteins were isolated from the extracts by methanol-chloroform precipitation and the rhodamine B removed by methanol washes, protein pellets were then resuspended in 100 μl of protein extraction buffer and protein concentrations calculated by Bradford reactions.

### Quantitative Reverse Transcription-Polymerase Chain Reaction

mRNA levels were measured by quantitative reverse transcription-polymerase chain reaction (qRT-PCR) as published (Schiessl et al., 2012), with primers 8–11 listed in Table 1.

## Results

Genes annotated as belonging to the pectin lyase superfamily are enriched among candidate targets of RPL (Bencivenga et al., 2016; Supplementary Table 1); among these, *PLL12* (*AT5G04310*) showed clear binding to RPL in three ChIP-seq biological replicates, but in none of the negative controls (Supplementary Figure 1). Furthermore, *PLL12* expression was located specifically in vascular cell types in a root transcriptomic atlas (Brady et al., 2007) and was associated with vascular differentiation *in vitro* (Kondo et al., 2016). Based on these data, we hypothesised that *PLL12* might have a role in cell wall modification during vascular development, in addition to the previously described function in stomatal guard cells (Chen et al., 2021).

**FIGURE 1:**
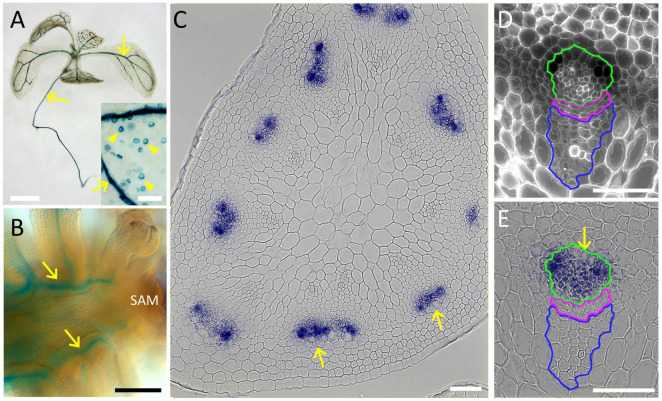
PLL12 is expressed in the stem phloem. **(A,B)**: *pPLL12:PLL12:GUS* seedling **(A)** and inflorescence apex **(B)** stained for GUS and cleared with chloral hydrate, showing GUS activity in the vasculature (arrows); in panel **(A)**, note also the dotted signal on cotyledons due to expression in stomatal guard cells. Bars: 1 cm **(A)**, 500 μm **(B)**. **(C–E)**: Cross-section of the inflorescence stem showing *pPLL12:PLL12:GUS* expression in phloem cells; **(C)**: overview of a stem section, with GUS staining in part of the vascular bundles (arrows); **(D)**: calcofluor-stained section through a single vascular bundle, with the phloem, cambium and xylem regions enclosed in green, magenta, and blue lines, respectively; **(E)**: same section as in panel **(D)**, showing GUS staining specifically in the phloem (arrow); bars: 50 μm.

To confirm the *PLL12* expression during vascular development, we used the GUS reporter fused to the complete *PLL12* gene. Previous reporter genes using GUS fused to 2 kb of the putative *PLL12* promoter gave weak and variable expression (Sun and Van Nocker, 2010) or suggested widespread expression (Chen et al., 2021). Our ChIP-seq data, however, indicated RPL binding to intron 3 of *PLL12* (Supplementary Figure 1), raising the possibility that regulatory sequences have been missed in previous constructs. To include all potential regulatory sequences, we used a genomic fragment that fully complemented the *pll12* mutation (see below), with GUS fused in frame after the last exon of *PLL12*. In nine independent lines, this p*PLL12:PLL12-GUS* reporter was expressed in stomata and throughout the vasculature of seedlings (Figure 1). In both the root and in the inflorescence, expression initiated close to the apical meristem, indicating that *PLL12* begins to function during early stages of vascular development. Cross-sections of the stem showed that within the vascular strands, *PLL12* was expressed primarily in the phloem. This localised expression pattern was in agreement with the reported role of *PLL12* in stomatal guard cells (Chen et al., 2021) and with reports of phloem-specific *PLL12* expression in the root (Brady et al., 2007; Kalmbach et al., 2022) and during *in vitro* differentiation (Kondo et al., 2016).

**FIGURE 2:**
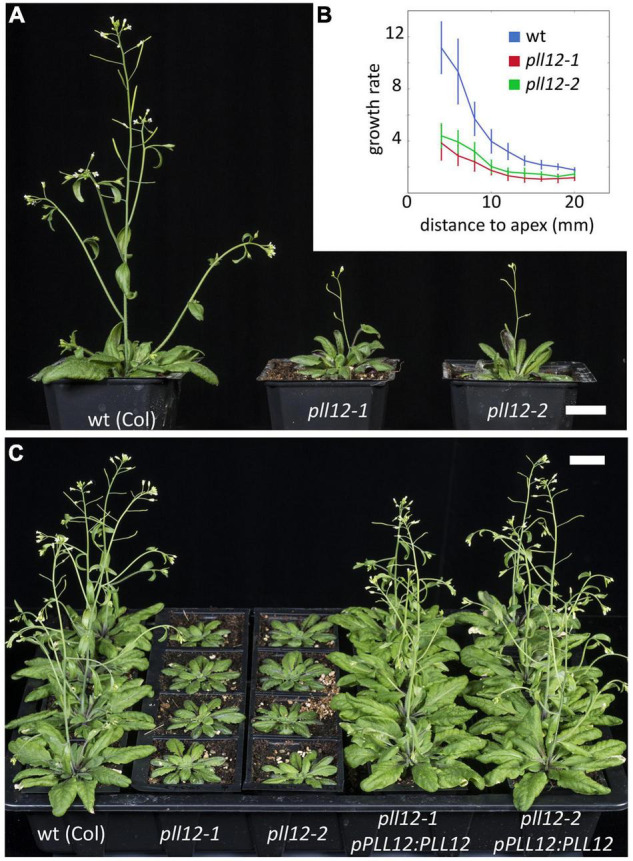
*pll12* mutants have reduced growth and slow stem elongation. **(A)**: Adult wild type (Col-0) and two different *pll12* mutant alleles grown side by side for 40 days. Both mutant lines bolted late and produced slow growing stems with few lateral organs. Scale bar: 2 cm. **(B)**: Relative growth rate (fold increase in length over 4 days) in Col-0 and *pll12* mutants measured at different starting positions along the stem. The stem elongates faster nearer to the apex and in the mutants this differential growth was significantly reduced. **(C)**: A genomic *pPLL12:PLL12* construct transformed into both mutant lines rescued the wild type phenotype. Scale bar: 2 cm.

We next analysed the function of *PLL12*, focussing especially on vascular development. For this, we used two independent T-DNA mutant alleles, *pll12-1* (SAIL 1207_A07) (Chen et al., 2021) and *pll12-2* (SAIL 1149_C06). As reported, *pll12-1* had undetectable *PLL12* mRNA (Chen et al., 2021), and the same was seen for *pll12-2* (Supplementary Figure 2). Both alleles caused similar phenotypes: the mutants were dwarf and late flowering, with slow stem growth (Figure 2). Complementation with full genomic fragments (from 1,884 bp upstream of the start codon to 702 bp downstream of the stop codon) confirmed that the *pll12* mutations caused these phenotypes, and that the selected genomic region contained all regulatory and coding regions required for function (Figure 2). Because the mutants had comparable and fully recessive phenotypes, from this point on we used the previously characterised *pll12-1* mutant.

**FIGURE 3:**
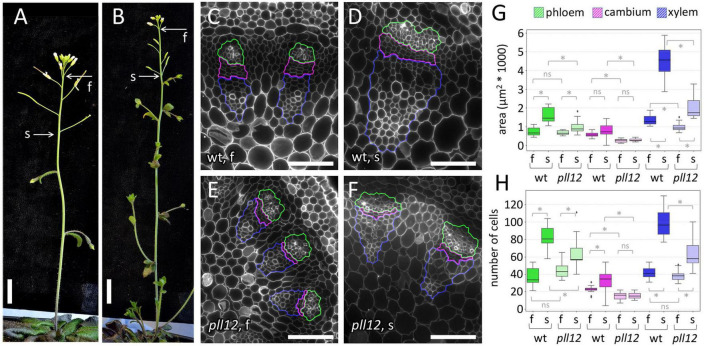
PLL12 is required for growth of the cambium and vascular bundles in the stem. **(A,B)**: overview of wt **(A)** and *pll12-1* **(B)** inflorescence stems, with the position of the most mature flower before organ abscission **(f)** and of the oldest silique (s) indicated; scale bars: 1 cm. **(C–F)**: calcofluor-stained cross-sections of vascular bundles of wt **(C,D)** and *pll12-1* **(E,F)** stems, at the position of the most mature flower **(C,E)** or of the oldest silique **(D,F)**; the phloem, cambium and xylem regions are enclosed in green, magenta, and blue lines, respectively; scale bars: 50 μm. **(G,H)**: boxplots of the area **(G)** and cell number **(H)** within phloem, cambium and xylem regions selected as in **(C–F)**, comparing vascular bundles at positions f and s in wt and *pll12-1* stems; * and *ns* indicate significant and non-significant differences in pairwise comparisons (*p* < 0.05, Mann-Whitney test); Supplementary Table 2 contains a full statistical analysis, including numbers of replicates, *p*-values and power analysis.

The vascular pattern of *pll12-1* appeared normal and was uninterrupted in leaves and in the inflorescence apex (Supplementary Figure 3). The vascular bundles contained all the expected cell types in their correct positions, but appeared smaller, especially in the mature region of the inflorescence stem (Figure 3). To detect growth defects, we measured the size and cell numbers in different regions of the stem vasculature. To compensate for differences in the growth rate between the mutants and the wild type, stems were collected when the fifth flower had matured (i.e., after a longer period of growth in *pll12-1*). Within stems at this stage, we collected sections at two positions: at the base of the fifth flower, corresponding to the immature region of the stem, which is still elongating (less than 1 cm of the apex, see Figure 2), and near the first silique, where stem elongation is complete and the vascular bundles have started to deposit secondary cell walls (more than 2 cm from the apex, Figure 2). In *pll12-1*, the size of immature vascular strands was not different from the wild type (number of cells per cross-section, mean ± SD: 101.4 ± 19.9 in wt, 98.7 ± 15.9 in *pll12-1*, Supplementary Table 2). However, as the stem matured, differences became clearer (213.9 ± 27.7 in wt, 142.4 ± 33.5 in *pll12-1*, Supplementary Table 2) and, contrary to the expectation based on the expression pattern, were not specific to the phloem. Instead, all three regions of the vascular strand (phloem, cambium and xylem) had reduced growth, measured either by cross-sectional area or cell numbers (Figures 3D,F–H). The region most affected was the cambium, which already had significantly fewer cells in the mutant before differences were detectable in the phloem and xylem (Figures 3G,H). Furthermore, the cambium was the only region with no detectable growth (measured as either cross-sectional area or by number of cells in cross-section), in contrast to the phloem and xylem, which grew at about half the wild-type rate. These results showed that *PLL12* is not essential for the initial vascular patterning or differentiation but is required for subsequent growth of all regions of the vascular strand, particularly the cambium.

**FIGURE 4:**
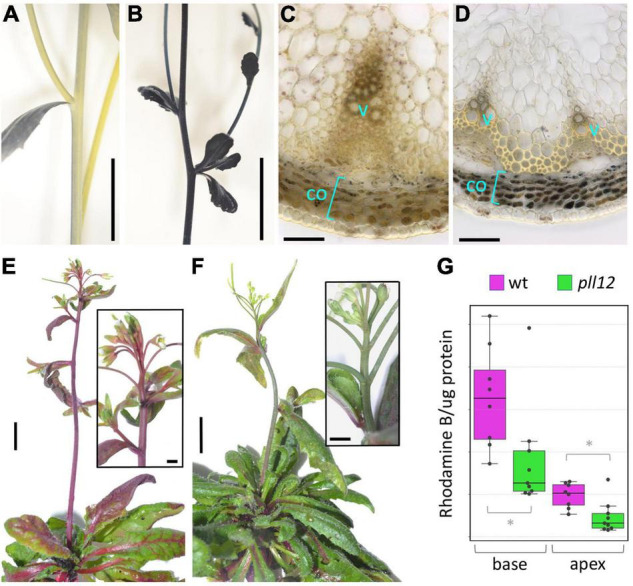
PLL12 is required for both phloem and xylem function. **(A,B)**: inflorescence stems of wt **(A)** and *pll12-1* **(B)** stained with Lugol’s solution, showing accumulation of starch in the mutant; scale bars: 1 cm. **(C,D)**: transversal sections of wt **(C)** and *pll12* **(D)** stems stained with Lugol’s solution, showing accumulation of starch grains in cortex cells (co) in the mutant; scale bars: 50 μm. **(E,F)**: wt **(E)** and *pll12-1* **(F)** plants grown for 20 h with their roots immersed in rhodamine B solution; the dye was transported efficiently throughout the wt inflorescence, whilst the mutant stems transported far less dye to the apex; insets show the inflorescence apices at a higher magnification; scale bars: 1 cm (main panels), 250 μm (insets). **(G)**: Boxplots showing Rhodamine content (normalised to protein concentration to correct for differences in tissue mass) in the apical and basal 1 cm segments of wt (magenta) and *pll12-1* (green) stems; * indicate significant differences (*p* < 0.05, Mann-Whitney test; full statistical analysis in Supplementary Table 2).

To reveal physiological consequences of the histological defects described above, we investigated whether *pll12-1* affected phloem and xylem function. Using carboxyfluorescein diacetate (CFDA) as a tracer, reduced phloem transport from hypocotyls to roots was shown in *pll12* seedlings (Kalmbach et al., 2022). Although we were not able to adapt this method to measure phloem transport in the inflorescence stem, it would be reasonable to assume that a similar defect would occur in the stem vasculature. As an indirect method to detect defects in phloem function in the stem, we assayed for high starch levels after a dark period, which are typically seen in plants with compromised sugar transport (Barratt et al., 2010; Bezrutczyk et al., 2018). Consistent with a defect in sugar export, staining with Lugol’s Iodine reagent revealed a strong accumulation of starch in the cortex of the inflorescence stem in *pll12-1* compared to the wild type (Figure 4). To assess xylem function, we measured transport of rhodamine B dye from roots to the inflorescence apex. Both visually (Figures 4E,F) and based on the dye concentration in the apex (normalised to protein concentration, Figure 4G), the mutant showed a clear reduction in transport through the xylem. We conclude that the reduced growth of vascular bundles in *pll12* was accompanied by a decrease in both sugar and water transport, which might be the cause of the general inhibition of growth in the mutant. Similarly, slow growth of the cambium and xylem in the mutant might be an indirect consequence of a reduced sugar supply, caused by a primary defect in the phloem. Alternatively, *PLL12* might be required to produce a phloem-derived signal that promotes development of the cambium and xylem.

**FIGURE 5:**
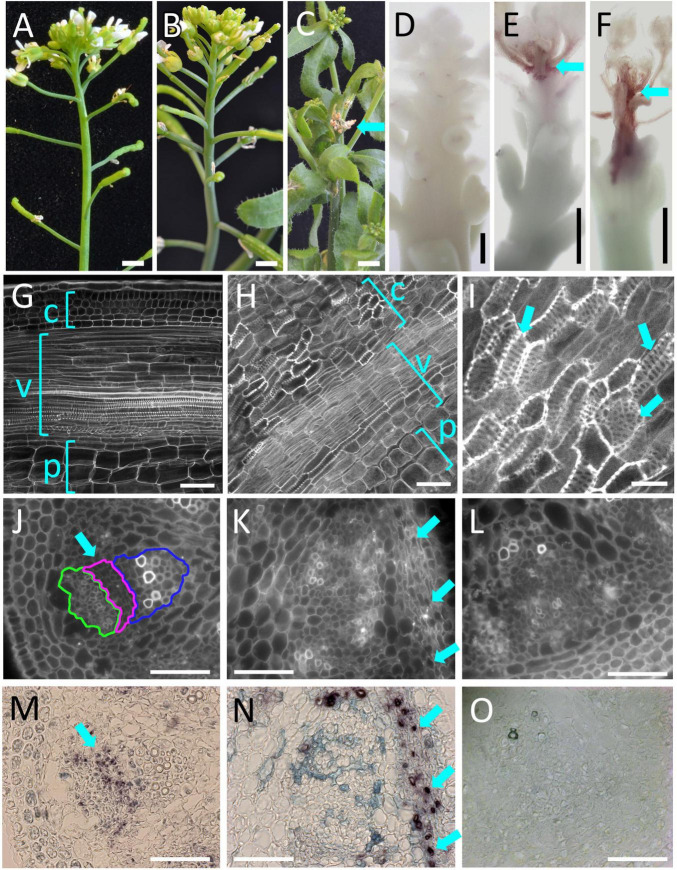
*PLL12* over-expression induces ectopic xylem differentiation and expression of a cambium marker. **(A–C)**: Mock-treated *RPS5A:LhGR,pOp:PLL12* **(A)** looked normal, whilst induction of PLL12 expression with dexamethasone resulted in mild [**(B)**, shortened internodes] to severe effects [**(C)**, with death of the stem apex (arrow) and lateral organs]; scale bars: 2 mm. **(D–F)**: Mock-treated **(D)** or dexamethasone-treated **(E,F)** inflorescence apices of *RPS5A:LhGR, pOp:PLL12* plants stained with phloroglucinol, showing mild **(E)** to severe **(F)** lignification; scale bars: 400 μm. **(G–I)**: Confocal images of *RPS5A:LhGR:PLL12* stem apices stained with mPS-PI after mock-treatment **(G)** or dexamethasone treatment **(H,I)**; c, v, and p indicate cortex, vasculature and pith; **(I)** higher magnification of stem section similar to panels **(H)**, with arrows indicating cells with the pitted walls characteristic of xylem elements and the top left arrow marking an opening between adjacent pitted cells. Scale bars: 50 μm **(G,H)**, 20 μm **(I)**. **(J–O)**: *In situ* hybridisation experiments showing PXY expression after mock **(J,L,M,O)** or dex-treatment **(K,N)** of *RPS5A:LhGR:PLL12* stem apices. All sections were incubated with PXY anti-sense probe except **(L,O)** (sense probe controls). **(J–L)** calcofluor images of the corresponding images shown in panels **(M–O)** respectively; in panel **(J)**, phloem, cambium and xylem regions are enclosed in green, magenta and blue lines, respectively. The normal *PXY* expression pattern in the cambial and early xylem precursor cells [arrows in panels **(J,M)**] is disrupted after dexamethasone treatment, which also induces strong *PXY* expression in a subset of cortical cells [arrows in panels **(K,N)**]. Bars: 50 μm.

To test whether increased or ectopic *PLL12* expression might be sufficient to promote cambium and xylem development, we generated plants in which *PLL12* transcription could be induced ubiquitously. To do this, we used an *pOp:PLL12* construct expressed in an *RPS5A:LhGR* background; when tissues are exposed to dexamethasone, this system induces *PLL12* expression under the widely expressed *RIBOSOMAL SUBUNIT 5A* promoter (Supplementary Figure 4). After inflorescence tips were treated five times at 2-day intervals, dexamethasone-treated plants showed variable inhibition of growth, ranging from short internodes to death of the shoot apex (Figures 5A–C). These phenotypes were associated with different levels of lignin deposition in the cortex and vascular bundles within 1–2 mm below the shoot meristem (Figures 5D–F). In apices with a mild phenotype, it was possible to trace the earliest deposition of ectopic lignin to strands of cells leading basally away from the shoot apical meristem—this position is typically occupied by procambial strands in the wild type (Supplementary Figure 5), suggesting that cells at early stages of vascular differentiation were particularly sensitive to ectopic PLL12 expression.

Ectopic lignification is one of the responses elicited by cell wall fragments during pathogen attack (Ferrari et al., 2013), so lignification could have been a defence response activated by pectin fragments produced when PLL12 was overexpressed. However, closer examination of the dexamethasone-treated apices revealed cortex cells with lignification in a pitted pattern and openings between adjacent cells (Figure 5I), similar to root metaxylem and to ectopic metaxylem cells induced by the regulators of xylem differentiation, VND6 and SND5 (Figures 5G–I; Kubo et al., 2005; Zhong et al., 2021). Pitted cells were also seen within the vascular bundles of plants with a mild phenotype after PLL12 induction, but not in uninduced controls (Supplementary Figure 5). Further supporting the idea that ectopic *PLL12* expression re-directed cortex cells to vascular development, ectopic expression of the regulator of cambium development, *PXY*, was detected by *in situ* hybridisation after PLL12 induction (Figure 5N). Conversely, the normal *PXY* expression in the cambium of the wild type was disrupted in the disorganised vascular bundles seen after dexamethasone treatment (Figures 5J–O). Together with the inhibition of cambium and xylem growth in the *pll12-1* mutant (Figure 3), the results above supported a role for *PLL12* in the regulation of cambium and xylem development, although the experiments could not distinguish whether *PLL12* promoted cambium and xylem identity sequentially, or separately in different cells.

**FIGURE 6:**
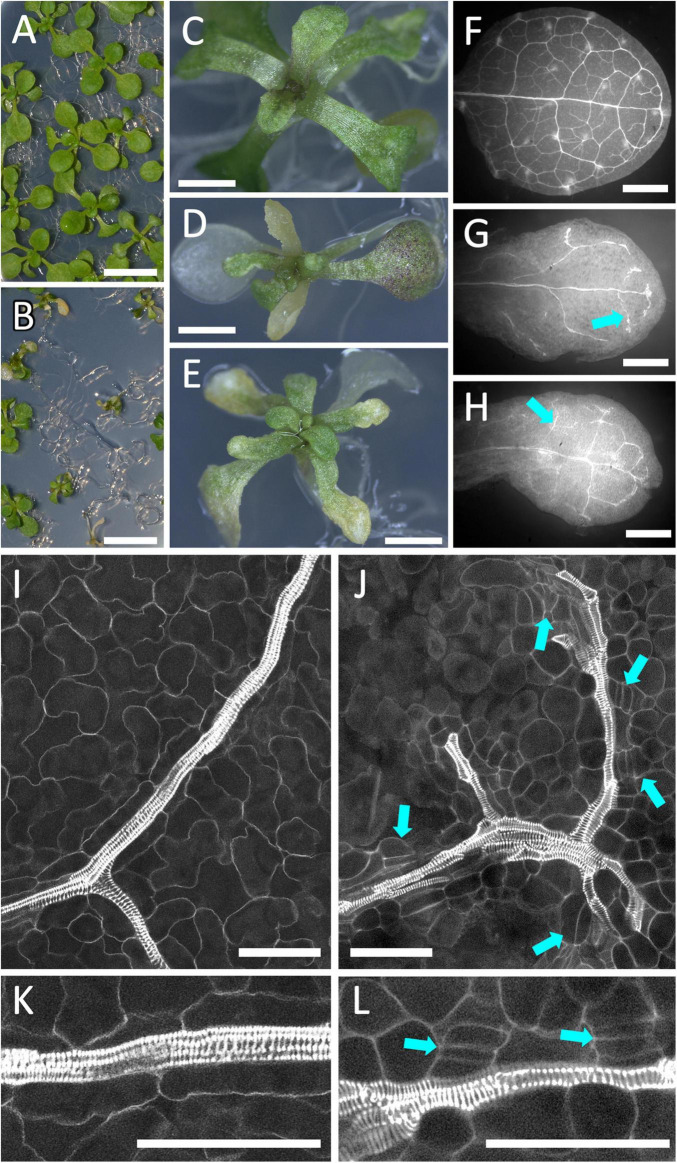
Over-expression of *PLL12* disrupts the development of leaf vasculature. **(A–E)**: *pRPS5A:LhGR pOp:PLL12* plants growing for 4 days on medium containing dexamethasone **(B,D,E)** showed reduced growth and leaf chlorosis, in contrast to mock-treated plants **(A,C)**; scale bars: 5 mm **(A,B)**, 2 mm **(C–E)**. **(F–H)**: Low magnification images of mPS-PI-stained leaves from an uninduced control **(F)** and two seedlings grown for 4 days on dexamethasone-containing medium **(G,H)**; arrows indicate interrupted, uneven vasculature seen after PLL2 induction; scale bars: 200 μm. **(I–L)**: Confocal images of leaf veins from seedlings comparable to panels **(A–H)**, grown without **(I,K)** or with dexamethasone **(J,L)**; panels **(K,L)** are higher magnifications of panels **(I,J)**; arrows indicate cell divisions oriented parallel to the veins, in a cambium-like pattern; scale bars: 50 μm.

The stronger effects of *PLL12* overexpression close to the meristem and in cortex cells suggested that the response to a putative PLL12-produced signal depended on developmental stage and cell type. To study the effect of ectopic PLL12 in a different context, we also looked at seedlings. *RPS5A:LhGR* pOp:*PLL12* plants were grown on medium without dexamethasone until the first pair of leaves emerged, then the seedlings were moved on to dexamethasone or mock treatment plates. Over the subsequent 7 days, a gradient of phenotypes became evident, ranging from small rosettes to stunted seedlings with severely deformed leaves (Figure 6), although root growth was initially unaffected (Supplementary Figure 6). In the most extreme cases, leaf blades failed to expand and became chlorotic (Figures 6D,E). Confocal imaging of these leaves stained with mPS-PI showed that the veins had irregular thickness, were discontinuous and showed cambium-like cell divisions along the leaf vasculature (Figures 6F–L). Thus, although ectopic *PLL12* only appeared to induce extensive lignification in the inflorescence apex, it interfered with the differentiation of vascular cells both in the inflorescence and in seedlings.

## Discussion

*PLL12* has been shown to function in stomatal guard cells, where it affects cell wall properties and the speed of stomatal opening and closure (Chen et al., 2021). Here, we show that *PLL12* is also required for the development and function of the stem vasculature. Loss of *PLL12* function did not have obvious effects on patterning or cell differentiation, but inhibited growth of vascular bundles, with the earliest effects visible in the fascicular cambium, followed by reduced cell numbers in both phloem and xylem. The reduced cell numbers throughout the stem vascular bundles were accompanied by reduced transport of both water and sugar. The simplest interpretation of these phenotypes is that *PLL12* function is limiting for the activity of the fascicular cambium, and consequently for the enlargement of vascular bundles during stem growth.

Despite the reduced growth across the vasculature (Figures 3G,H and Supplementary Table 2), *PLL12* expression was specific to the phloem, in line with previous reports (Brady et al., 2007; Kalmbach et al., 2022). Thus, the effects on cambium and xylem growth were likely indirect consequences of a primary role of *PLL12* in the phloem. In support of a phloem function for *PLL12*, a recent pre-print reports that *pll12-1* has a defect in long-distance phloem transport, attributed to subtle changes in the formation of sieve plates between phloem elements (Kalmbach et al., 2022). However, defects in long-distance sugar transport would not readily account for the disproportionate effect of *pll12-1* on the growth of established vascular bundles, in comparison to earlier stages. Moreover, a role exclusively in sieve plate formation would not easily explain why *PLL12* overexpression induced ectopic cambium and xylem features and disrupted vascular differentiation in seedlings. To explain both loss- and gain-of-function phenotypes, and taking into account the phloem-specific expression, we propose that in addition to the reported role in sieve element development (Kalmbach et al., 2022), *PLL12* activity influences an intercellular signal that coordinates development across vascular tissues.

PLL12 contains a canonical pectate lyase C domain, with conserved residues implicated in catalysis, Ca^ + +^ binding and exocytosis (Chen et al., 2021), so this protein is likely to cleave demethylesterified pectin within the cell wall. Accordingly, immunolocalisation showed increased levels of calcium-crosslinked pectin in *pll12-1* stomatal walls (Chen et al., 2021) and mutation of the predicted pectate lyase catalytic site abolished the ability of *PLL12* to complement the mutant (Kalmbach et al., 2022). Furthermore, PLL12 is homologous to a *Zinnia* PLL protein for which pectate lyase activity has been demonstrated *in vitro* (Domingo et al., 1998). Pectin cleavage would be expected to release oligogalacturonides (OGs), which bind to the WAK1 receptor and activate the cell wall integrity and pathogen defence signalling pathways, whose downstream responses include lignification (Caño-Delgado et al., 2003; Brutus et al., 2010; Ferrari et al., 2013). However, the induction of metaxylem-like pitted cell walls, combined with ectopic *PXY* expression, suggested that lignification after ectopic *PLL12* expression was an aspect of vascular differentiation, rather than a generic defence or cell wall stress response.

Thus, the expected biochemical function of PLL12, together with its non-cell-autonomous effects and its ability to ectopically induce cambium and xylem identity, raise the possibility that a cell wall-derived signal participates in the regulation of vascular differentiation. The most straightforward candidate signal would be OGs, although their developmental roles remain speculative (Seifert and Blaukopf, 2010). Supporting a link to xylem differentiation, genes that respond early to OG treatment (Moscatiello et al., 2006) included *SND5*, which is part of the transcriptional network controlled by *PXY* (Smit et al., 2020) and encodes a NAC domain protein that regulates secondary wall deposition in the xylem (Zhong et al., 2021). However, indirect effects on other signalling pathways are also possible. External application of OGs has been shown to interfere with auxin signalling (Branca et al., 1988; Ferrari et al., 2013). However, the phenotypes caused by ectopic PLL12 activation were different from those seen in plants with an overall inhibition of auxin signalling; for example, there was little effect on roots, where auxin plays a central role (Israeli et al., 2020). The interrupted veins seen after ectopic PLL12 induction in cotyledons and leaves are also reminiscent of phenotypes seen after application or overexpression of the PXY ligand CLE41/44 (Hirakawa et al., 2008), or in mutants that affect phosphoinositide-regulated vesicle traffic, which may regulate auxin transport (Sieburth et al., 2006; Carland and Nelson, 2009). In the future, it will be interesting to explore how PLL12 function interacts with these signalling pathways.

## Data Availability Statement

The original contributions presented in the study are included in the article/Supplementary Material, further inquiries can be directed to the corresponding author.

## Author Contributions

RS: conceptualisation, software, funding acquisition, and supervision. MB and VS: investigation. MB and RS: writing—original draft. All authors: formal analysis, data curation, and writing—review and editing.

## Conflict of Interest

The authors declare that the research was conducted in the absence of any commercial or financial relationships that could be construed as a potential conflict of interest.

## Publisher’s Note

All claims expressed in this article are solely those of the authors and do not necessarily represent those of their affiliated organizations, or those of the publisher, the editors and the reviewers. Any product that may be evaluated in this article, or claim that may be made by its manufacturer, is not guaranteed or endorsed by the publisher.

## Footnotes

1
http://matplotlib.org

2
http://www.scipy.org
