# Supplementary material for: A Phloem-Expressed PECTATE LYASE-LIKE Gene Promotes Cambium and Xylem Development
Source: Front Plant Sci. 2022 Apr 26;13:888201. doi: 10.3389/fpls.2022.888201 (PMC9087803; doi:10.3389/fpls.2022.888201)
Supplement: Supplementary file 1 [file Data_Sheet_1.pdf]

Bush et al.

A phloem-expressed *PECTATE LYASE-LIKE* gene promotes cambium and xylem development

Supplementary figures and legends

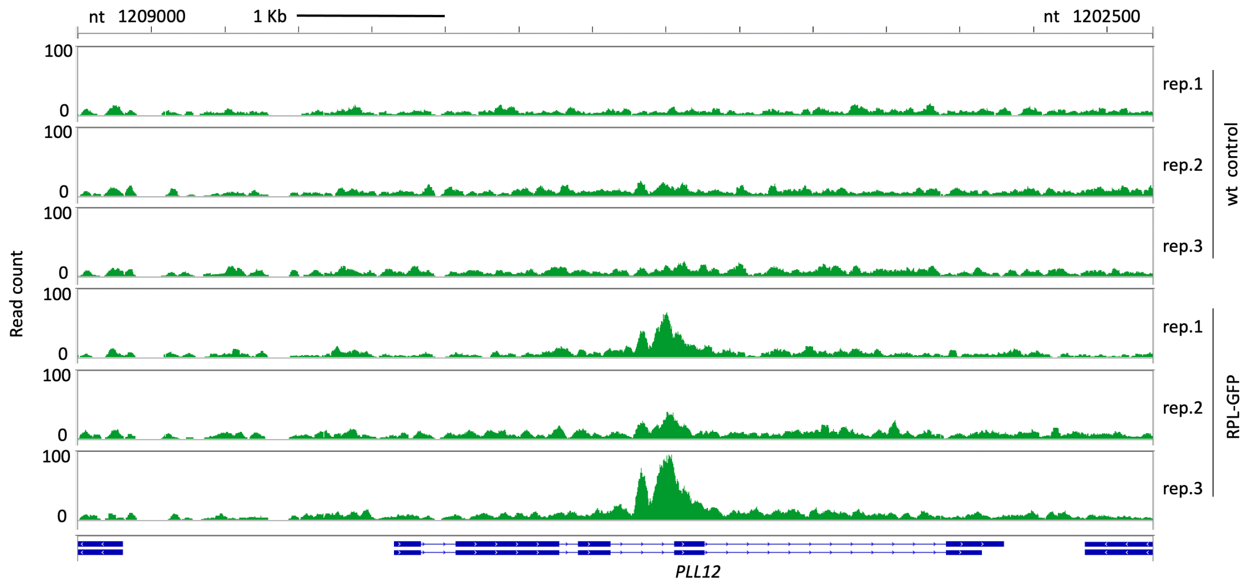

Figure S1: ChIP-seq evidence of RPL binding to *PLL12*

GFP-tagged RPL was used to pull down associated genomic DNA in inflorescence apices; full experimental details, data analysis and data access have been described [26]. Histograms of ChIP-seq reads (green) are shown for the genomic region containing the full *PLL12* gene and intergenic regions up to the adjacent genes. Each graph shows a biological replicate; wt control and RPL-GFP correspond, respectively, to plants without tagged RPL or expressing tagged RPL. The positions of exons and introns are indicated by blue bars and lines, respectively; nucleotide positions on chromosome 5 and scale in Kb are indicated above the graphs. Note the peaks around intron 3 of *PLL12*, specifically in the RPL-GFP replicates.

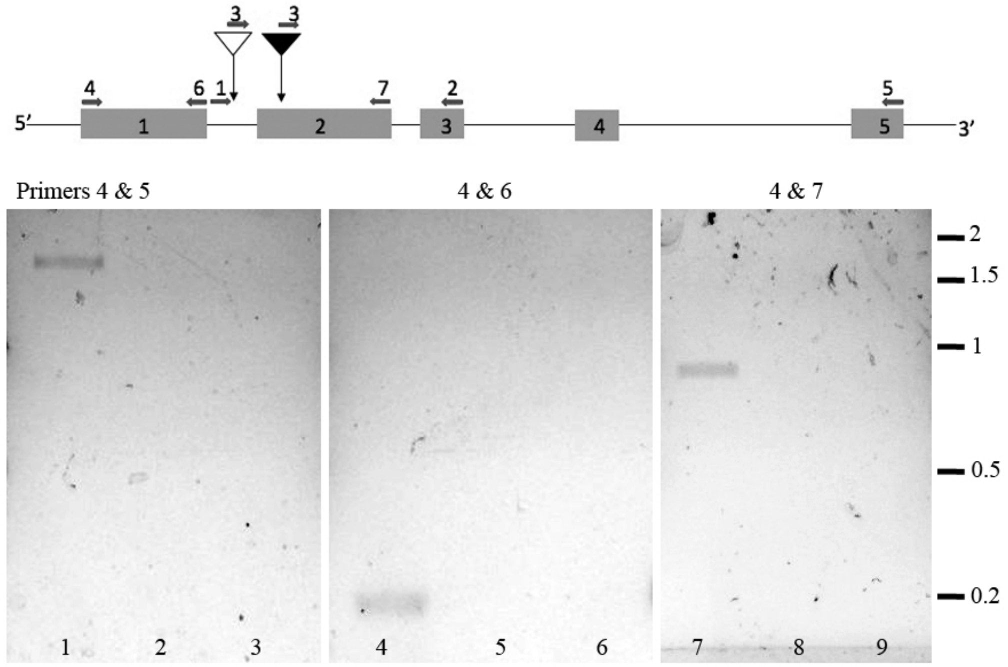

Figure S2: Loss of function *pll12* alleles.

A) Schematic of the *PLL12* gene showing the location of T-DNA insertions for *pll12-1* (white triangle, SAIL 1207_A07) and *pll12-2* (black triangle, SAIL 1149_C06); the numbered arrows represent primers used for genotyping and for amplifying cDNA (sequences listed in Table 1). Grey boxes represent exons 1-5 separated by introns (black lines). The gel image below shows RT-PCR products obtained with the primers indicated, using RNA from wild-type (lanes 1, 4, 7) and *pll12-2* (lanes 2, 3, 5, 6, 8, 9) seedlings. DNA size markers are shown on right (in Kb). Similar results were obtained with *pll12-1*, as reported [30].

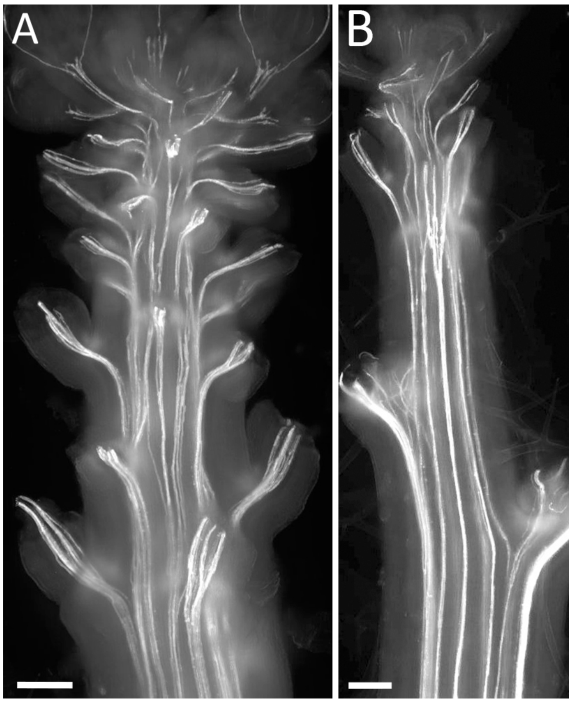

Figure S3: Vascular strands are uninterrupted in the *pll12-1* inflorescence apex.

A, B) Whole-mount images of wild-type (A) *and pll12-1* (B) stem apices stained with mPS-PI. Scale bars: 200 µm.

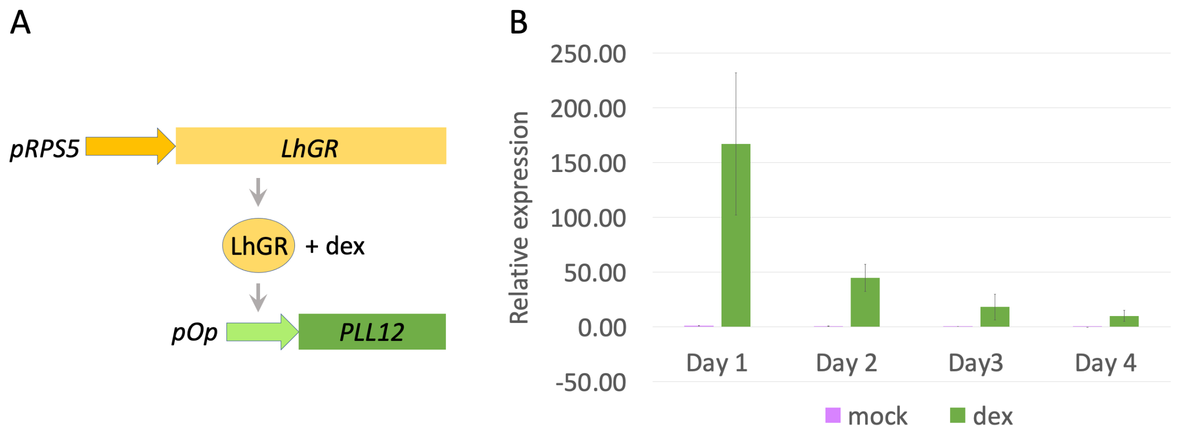

Figure S4: Inducible *PLL12* expression.

A) Diagrams of *RPS5A:LhGR* and *pOP: PLL12*; the *RPS5A* promoter drives ubiquitous expression of the dexamethasone-dependent, artificial transcription factor LhGR [57]; after dexamethasone treatment, LhGR activates *PLL12* expression from its target promoter, *pOp*. B) Expression of *PLL12* at different days after mock (magenta) or dexamethasone (green) treatment, measured by qRT-PCR at different times after dexamethasone treatment. Values were normalized to *TUBULIN4* as the internal standard and are expressed relative to the average value for mock-treated samples on day 1. Bars and lines show the average and standard deviation, respectively, for 3 to 4 biological replicates. The full raw and normalized data are shown in Table S2.

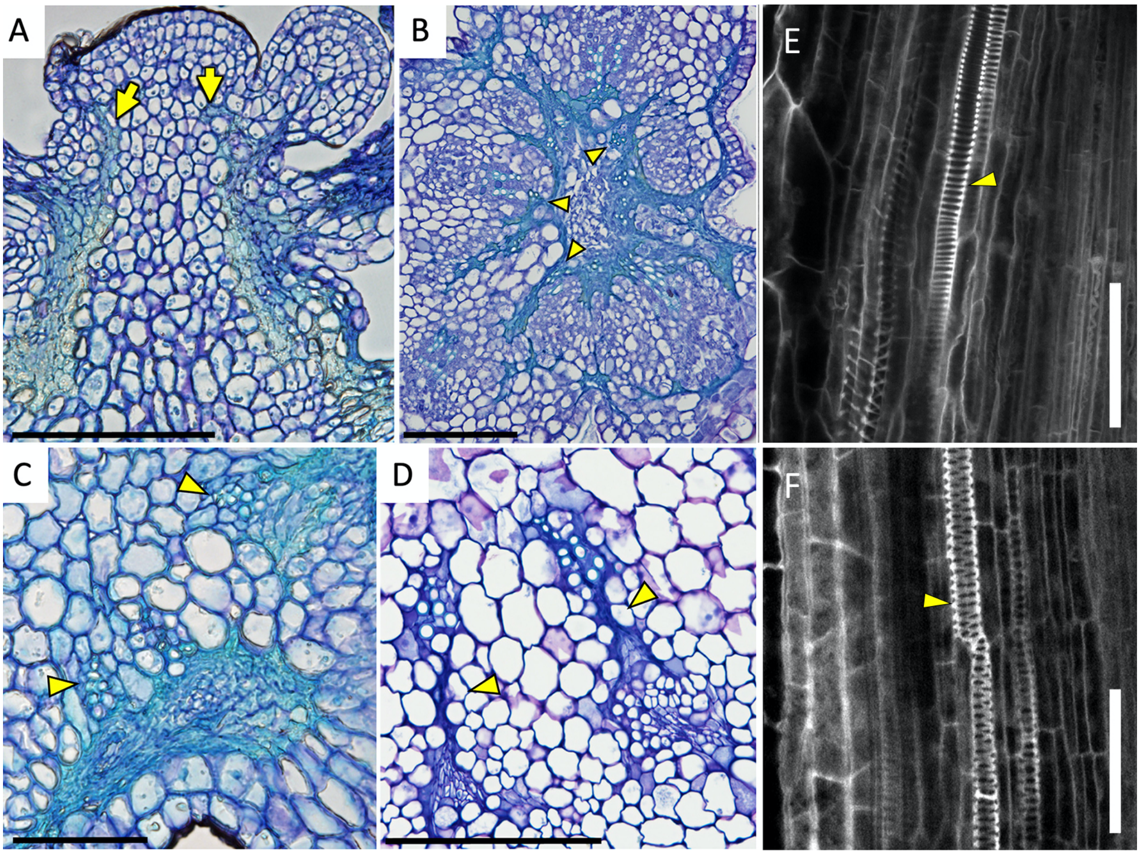

Figure S5: *PLL12*-induced lignification occurs preferentially along developing vascular strands, disrupts the organization of vascular bundles and induces metaxylem development.

A-D) The panels show toluidine-stained Technovit sections from dexamethasone-treated stem apices of *RPS5A:LhGR pOp:PLL12* plants. A) Lignin (cyan) is deposited in regions where the procambium normally lies (arrows) and extends away from the meristem to encompass the vascular bundles (B-D, arrowheads indicate the positions of bundles). During this process, the distinctive structure of the bundles is disrupted (C-D).
E-F) Confocal images of mPS-PI-stained vascular bundles in *RPS5A:LhGR pOp:PLL12* plants with mild phenotypes after dexamethasone treatment (F) compared to mock-treated control (E); arrowheads point to cell wall features characteristic of protoxylem (E) or metaxylem (F). Scale bars A, B, D: 100 um; C, E-F: 50 um.

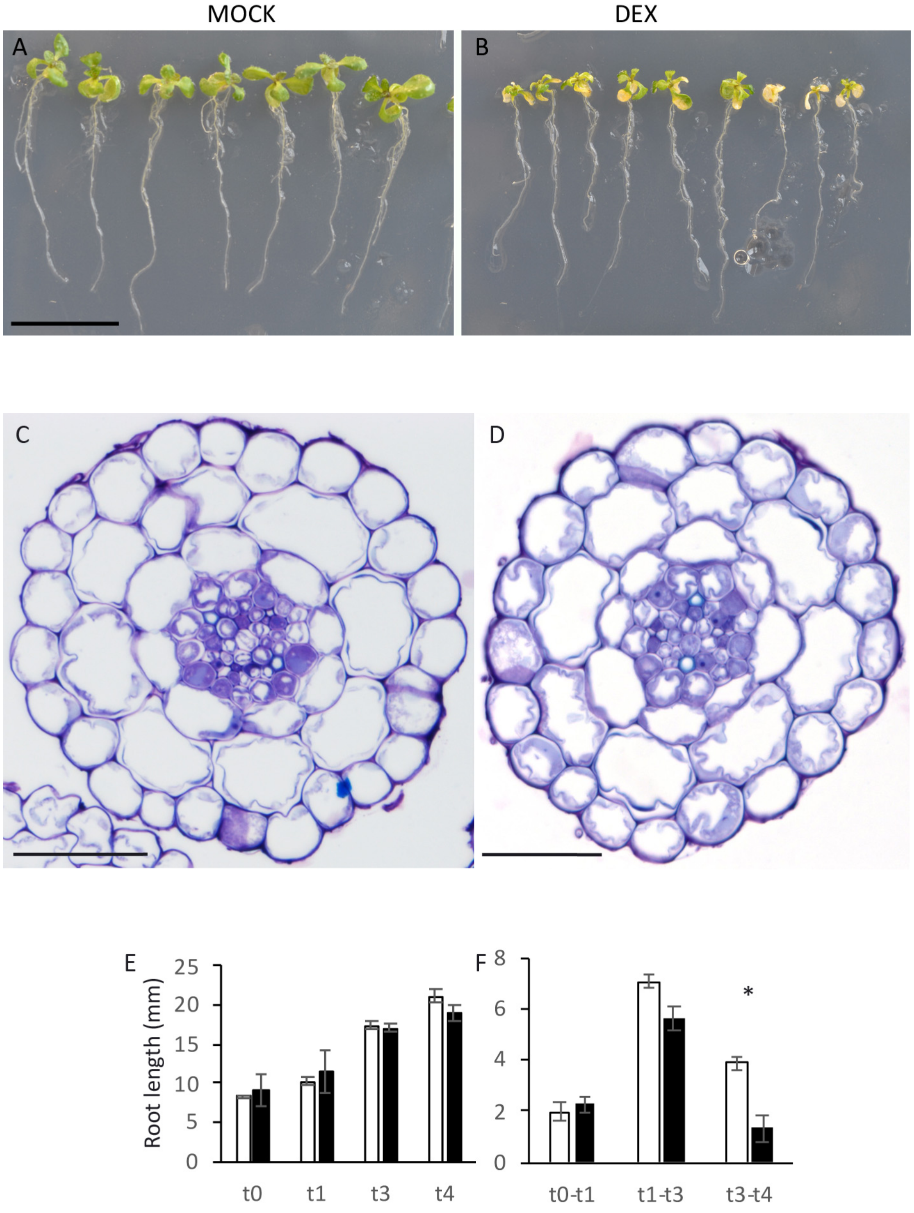

Figure S6: Over-expression of *PLL12* has no immediate effect of root growth.

A, B) *RPS5A:LhGR pOp:PLL12* seedlings grown on mock- (A) or dexamethasone-induction (B) media for 4 d; note the smaller rosette and chlorotic leaves after growth with dexamethasone.

C, D) Toluidine-stained resin sections of *RPS5A:LhGR pOp:PLL12* roots grown on mock- (C) or dexamethasone-induction (D) media; note that the histology was not visibly affected by *PLL12* induction.

E, F) Total root length was unaffected over the 4d growth period (mock, white bars, dexamethasone-induced, black bars), but once the leaves became chlorotic (t3-4)

incremental root elongation (F) started to be reduced by PLL12 over-expression; asterisk *t*-test P=0.02.

Scale bars: A-B, 1.5cm, C-D, 40 um.
